# Iron deficiency anemia associated factors and early childhood caries in Qingdao

**DOI:** 10.1186/s12903-022-02127-z

**Published:** 2022-03-31

**Authors:** Shuaiqi Ji, Xiaohang Guan, Lei Ma, Pingping Huang, Hao Lin, Rui Han

**Affiliations:** 1 Department of Stomatology, the Affiliated Hospital of Qingdao University, Wutaishan Road & 1677, Qingdao City, 266003 Shandong China; 2 School of Stomatology, Qingdao University, Qingdao, 266003 China; 3 Hexi Clinic of Tianjin Stomatological Hospital, Tian Jin, 300000 China

**Keywords:** Iron deficiency anemia, Early childhood caries, Iron intake

## Abstract

**Background:**

Iron deficiency anemia (IDA) has been shown to be related to early childhood caries (ECC). However, data on the relationship, if any, between IDA-associated factors and ECC remain scant. This study aimed to explore the interplay between IDA-associated factors and ECC.

**Methods:**

This study randomly sampled a total of 1598 children in Qingdao city, and analyzed the severity of ECC using decayed-missing-filled teeth index, while the rate of caries was analyzed following the WHO recommendations. The correlation between IDA and ECC was analyzed by both the chi-square test and Mann–Whitney U test. In addition, we designed an electronic questionnaire and employed the disordered multi-classification logistic regression to interrogate the relationship between the IDA-associated factors and ECC.

**Results:**

Children with IDA had higher rates and severe ECC than those without IDA (*p* < 0.001). Children who were breastfed until 2 years old had a higher risk of IDA and ECC, compared to those who were not {OR 3.453 (1.681–7.094)}. Compared with children who had no history of IDA at the age of 2 years or below, those with IDA history had a higher risk of IDA and ECC {OR 8.762 (3.648–21.041)}. In addition, children who had a maternal history of IDA at pregnancy were at a higher risk of IDA and ECC compared to those who had no IDA history at pregnancy {OR 4.913 (2.934–8.226)}. Our data showed that children from a family with an annual income lower than 50,000 Renminbi (RMB) had a higher risk of IDA and ECC compared to those with an annual family income higher than 200,000 RMB {OR 3.421 (1.505–7.775)}. On the other hand, compared with children taking iron supplements, children who did not were at a higher risk of ECC and IDA {OR 5.602 (1.858–16.896)}.

**Conclusion:**

Factors such as low family income, history of IDA in children aged 2 years or younger, IDA history during pregnancy, children breastfed until 2 years old, and those not taking iron supplements were significantly associated with the occurrence of ECC and IDA.

**Supplementary Information:**

The online version contains supplementary material available at 10.1186/s12903-022-02127-z.

## Background

Early childhood caries (ECC) remains one of the most common childhood diseases [1]. Besides, iron deficiency anemia (IDA), which is a severe stage of iron deficiency (ID), is highly prevalent in preschool children (< 5 years old) [2]. Previous cross-sectional studies have shown that children with anemia or IDA had a higher risk of caries than those without the deficiency [3, 4]. Other cross-sectional and case–control studies demonstrated that children with ECC were more likely to be iron deficient [5, 6]. In addition, children with ECC have lower levels of serum ferritin, serum iron, and hemoglobin [7, 8].

On the other hand, in vivo analyses showed that mice fed on iron supplements had lower rates of caries and severity [9, 10]. This was associated with iron resistance to caries. Iron has been shown to have three negative effects on *Streptococcus mutans* cariogenic potential: (1) suppression of enamel demineralization in acidic environments [11]; (2) reduction of dental plaque acidity [12]; (3) disinfectant and bacteriostatic ability with *S. mutans* [13]; as well as (4) inhibition of glycosyltransferase activity [14].

Data has shown that IDA is intrinsically related to ECC. Mechanistically, salivary gland functions are impaired in IDA, resulting in reduced salivary secretion and poor buffering capacity, which lead to inefficient wash out dental plaque and food debris, thus triggering dental caries [15]. In addition, there is reduction of ferric ions in saliva and blood during IDA [16]. Since iron has anti-caries features, it inhibits the activity of *S. mutans* virulence factors and creates a caries-prone environment. Dental caries results from interactions between bacteria, such as *S. mutans*, saliva components, and dietary carbohydrates, forming a biofilm that closely adheres to the teeth surfaces [17].

In addition, previous studies have demonstrated a mutual relationship between IDA and ECC. For instance, many children diagnosed with ECC may have inflammation in their primary tooth pulp, characteristic of necrosis, and the agony and discomfort can lead to changes in their chewing habits, decreased meat and fruit consumption, thus influencing the intake and supplementation of iron [18, 19]. This situation can lead to nutritional IDA. On the other hand, it has been suggested that treatment of dental caries would remove or relieve IDA without iron remedy [7]. This reinforces the argument that the relationship between ECC and IDA is associated with nutritional status. Chronic malnutrition in childhood life may harm alternate primary and permanent teeth, postponing eruption and exfoliation of milk teeth, and increasing the risk of caries in future [20]. However, whether the factors influencing body iron state could fuel the occurrence of ECC is yet to be determined. Here, we hypothesized that factors influencing body iron state may contribute to the development of caries.

## Methods

### Sample capacity and ethical approval

This cross-sectional investigation was conducted at the Qingdao Community Health Center, with the Affiliated Hospital of Qingdao University from April 1st to May 31st, 2019, in Qingdao. Qingdao is an advanced economy and a high population density city. This study followed the Statement of Strengthening the Reporting of Observational Studies in Epidemiology [21]. Ethical approval was given by the Ethics Committee of Affiliated Hospital of Qingdao University (QYFYWZLL 25,820), and written informed consent was obtained from parents or guardians for the minors who participated in the study. Age was calculated from the children’s birthday to the survey date. The sample capacity was calculated following the formula [22]:

$${\text{n}} = {\text{deff}}*\upmu _{{\upalpha /2}}^{2} *(1 - {\text{p}}){\text{p}}/\updelta ^{2}$$

n was the sample size, with deff 4.5. The level of confidence of μ was 1.96 when the confidence level for an accuracy of α was 0.05. The estimated rate was based on 70.9% of ECC in 5-year-old children according to the 4th National Oral Health Survey [23]. The allowable error, (δ), was assumed to be 5%, so the minimum sample was around 1570, after considering 10% of non-response rate.

We used a multistage, stratified, and random cluster sampling procedure to select the study sample. First, two districts (Shinan District, Shibei District) in Qingdao city were randomly selected using Probabilistic Proportional Sampling (PPS). Thereafter, six public and private kindergarten schools were distributed in each district. We then recruited 150 preschoolers 4, 5, and 6 years old from the selected kindergartens using the quota sampling method.

We included participants who did not use hormones or immunity inhibitors in the last half a year; (2) did not take antibiotics in the past month; (3) those with no major systemic diseases and (4) children who were brought up by their parents. Uncooperative families were excluded.

### Dental caries examination

After tooth cleaning and drying, oral examination was performed under natural light with a disposable planar endoscope and a probe. Three trained and licensed dental doctors performed the examination, following diagnostic criteria of the 4th National Oral Epidemiological Survey following the World Health Organization (WHO) guidelines [23]. Decayed-missing-filled teeth (dmft) index was also recorded. Here, "decayed" refers to the decayed and unfilled teeth, "missing" refers to the loss of tooth due to caries, and "filled" refers to the tooth with filling due to caries. At the end of each daily oral examination by three dental doctors, 5% of all inspection objects were randomly selected for repeated tests by two dental assistants every day. Repeat checks were carried out in agreement to inter and intra-examiners. Any potential bias was handled by discussions between the doctors and assistants.

### Blood sample collection and diagnostic criteria for IDA

1–2 mL of venous blood was collected from children on fasting by two community nurses and then put into EDTA-K2 anticoagulant tubes. Hemoglobin content and mean red blood cell volume were analyzed by an automatic hematology analyzer.

According to the WHO diagnostic criteria [24], children aged 5–59 months or 5–11 years are diagnosed with IDA when their hemoglobin content is lower than 110 g/L or lower than 115 g/L, respectively, and their mean red blood cell volume is lower than 75 fL.

### Questionnaire survey

Before inspection, we informed the parents of the purpose and method of examination, who signed informed consent. The electronic questionnaire was adapted to potential IDA influencing factors [25], and the study was accomplished in three days. Alpha reliability coefficients and KMO values of the questionnaires were more than 0.7. The questionnaires involved six IDA-associated factors, which included IDA history during pregnancy, annual family income, frequency of children's meat intake, IDA history at age 2 or below, children's intake of iron supplements, and breastfeeding within 2 years of age (see Additional file 1).

### Statistical methods

We exported and organized the data in Excel, and then SPSS 22.0 statistical software was used for data processing. The intra- and inter-examiner reliability was assessed by the Intraclass Correlation Coefficient (ICC). Correlation between IDA and ECC was analyzed by the chi-square test and the Mann–Whitney U test. Dependent variables were divided into four levels: ECC + positive IDA; caries-free + positive IDA; ECC + negative IDA; or caries-free + negative IDA. On the other hand, independent variables included duration of breastfeeding until 2 years old; maternal history of IDA during pregnancy; annual family income; frequency of meat intake; history of IDA in children ≤ 2 years old; and intake of iron supplements. Multivariate analysis was performed by disordered multiple classification logistic regression.

## Results

A total of 1,646 preschool children participated in the survey, and 1598 were included in the final analysis. The excluded 18 children were ill-matched, who did not cooperate in oral examination and blood collection. In addition, 30 families who could not complete the electronic questionnaire were also excluded. The intra- and inter-examiner’s reliability was excellent, with an ICC of above 0.90.

### Characteristics of the population and sociology

As shown in Table 1, there were a total of 1598 children, who included 854 boys and 744 girls. Children aged 4 years accounted for 37.9% of all the children, those aged 5 years were 35.2%, while those aged 6 years accounted for 26.9%. The proportion of children with a maternal history of IDA during pregnancy, IDA history at age 2 or below, breastfeeding until 2 years old, intake of iron preparations or IDA accounted for 18.3%, 6.6%, 9.1%, 11% or 5.8%, respectively. Household income was divided into four levels, with majority of the families at middle income level. On the other hand, frequency of the children’s meat intake was divided into three levels, with most of the children eating meat once to three times a week.

**Table 1:** Characteristics of population and sociology

	Variables	Numbers	Frequency (%)
Sex	Boys	854	53.4
	Girls	744	46.6
Age	4 years old	605	37.9
	5 years old	562	35.2
	6 years old	431	26.9
IDA history during pregnancy	Yes	292	18.3
	No	1306	81.7
Annual family income (ten thousand RMB)	0~	262	16.4
	5~	517	32.4
	10~	529	33.1
	20~	290	18.1
The frequency of children's meat intake	Rarely or never	184	11.5
	Once to three times a week	831	52
	Once a day or more	583	36.5
IDA history at age 2 or below	Yes	105	6.6
	No	1493	93.4
Duration of breastfeeding up to age 2	Yes	146	9.1
	No	1452	90.9
Children's intake of iron supplements	Yes	176	11
	No	1422	89
Iron deficiency anemia	Yes	93	5.8
	No	1505	94.2

### Relationship between IDA and ECC

Our analysis showed that children with IDA had higher rates of caries and dmft index (*p* < 0.001), as shown in Table 2.

**Table 2:** Association between iron deficiency anemia (IDA) and early childhood caries(ECC)

		Total	ECC (%)	*P*	dmft *M* (*P*_*25*_*,P*_*75*_)	*P*
IDA	Yes	93	75 (80.6)	< 0.001	3 (1,7)	< 0.001
	No	1505	875 (58.1)		1 (0,4)	

### Relationship between IDA-associated factors and ECC

Our data demonstrated that compared to children who were not breastfed before 2 years old, children who were breastfed until the age of 2 had a higher risk of IDA and ECC, or only IDA, with an odds ratio (OR) of 3.453 (1.681–7.094) and 1.697 (1.146–2.511), respectively. Unlike those who had no IDA history of at age 2 or below, children with IDA history had a higher risk of both IDA and ECC, only ECC, or only IDA, with an OR of 8.762 (3.648–21.041), 24.655 (7.745–78.486) or 4.502 (2.469–8.210), respectively. Besides, children who had IDA history during pregnancy were at a higher risk of IDA and ECC, compared to those who did not, with an OR of 4.913 (2.934–8.226). In addition, compared to those with an annual family income of higher than 200,000 Renminbi (RMB), children from a family with an income lower than 50,000 RMB had a higher risk of IDA and ECC, or only IDA, with an OR of 3.421 (1.505–7.775) or 1.630 (1.129–2.353), respectively. Similarly, compared to children taking iron supplements, children who did not were at a higher risk of IDA or ECC and IDA, with an OR of 1.585 (1.127–2.229) or 5.602 (1.858–16.896), as shown in Table 3.

**Table 3:** The relationship between IDA-associated factors and ECC

	Wald (x^2^)	*P*	OR (95% CI)
**IDA and ECC**			
Duration of breastfeeding up to age 2	11.379	**0.001**	3.453 (1.681–7.094)
The history of IDA at age 2 or below	23.576	**< 0.001**	8.762 (3.648–21.041)
IDA history during pregnancy	36.634	**< 0.001**	4.913 (2.934–8.226)
Annual family income			
Less than 50,000 RMB	8.620	**0.003**	3.421 (1.505–7.775)
50,000 to 100,000 RMB	0.299	0.585	1.243 (0.570–2.713)
10,0000 to 200,000 RMB	*0.225*	0.635	1.210 (0.551–2.653)
More 200,000 RMB			1
Children's no intake of iron supplements	9.361	**0.002**	5.602 (1.858–16.896)
**Only ECC**			
The history of IDA at age 2 or below	29.431	**< 0.001**	24.655 (7.745–78.486)
**Only IDA**			
Duration of breastfeeding up to age 2	6.977	**0.008**	1.697 (1.146–2.511)
The history of IDA at age 2 or below	24.097	**< 0.001**	4.502 (2.469–8.210)
Annual family income			
Less than 50,000 RMB	6.798	**0.009**	1.630 (1.129–2.353)
50,000 to 100,000 RMB	5.774	**0.016**	1.450 (1.071–1.963)
10,0000 to 2000,00 RMB	4.323	**0.038**	1.373 (1.018–1.851)
More 200,000 RMB			
Children's no intake of iron supplements	7.010	**0.008**	1.585 (1.127–2.229)

The group of caries-free + negative IDA was used as the reference group

Bold values indicate significant statistical differences (*P* < 0.05)

## Discussion

This study demonstrated that the rate of ECC in children from Qingdao was 59.4%, which shows that the caries prevalence among the children is still quite high. Thus, the families and government should pay attention to the effects of ECC on children, and initiate early interventions on factors fueling ECC.

The prevalence and severity of ECC was higher in children with IDA compared to those without the disease. This phenomenon was associated with the functions of salivary glands which is impaired IDA, leading to reduced salivary secretion and low buffering capacity. Consequently, there is ineffective washing of dental plaque and food residues, which easily leads to dental caries [15]. In addition, the interplay between ECC and IDA was due to decreased iron ions in saliva, which inhibit the activity of *S. mutans* and promote the formation of cariogenic biofilms when the body is in an iron deficient state [13, 16].

In sync with previous studies, our data showed that lower family annual income was associated with IDA [26, 27]. This could be because families with low income might not live a healthy life which include intake of iron-rich foods or lack immunity to combat infections, which increases the probability of having IDA [26]. However, the data showed that lower household income was not significantly associated with rate of ECC but was a risk factor for the co-occurrence of IDA and ECC. This might be due to suppressed systemic immune functions when the body is in an iron-deficient state, thus a higher susceptibility to cariogenic bacterial infection [28].

About half of the childbearing age women suffer from IDA [2], and the occurrence of IDA during pregnancy as well as pregnant women who do not have a scientific and reasonable diet plan have a higher risk for IDA. If a mother does not have IDA during pregnancy, her iron can meet the needs of a 4 or 5 months baby [29]. Otherwise, reduced maternal iron reserves and insufficient iron transmission from the placenta to the fetus increase children’s IDA risk. However, our study demonstrated that there is no correlation between maternal anemia during pregnancy and IDA occurrence in children aged 4 to 6 years old. This may be related to acquisition of iron from food. The development of children's primary teeth starts from the sixth week of an embryo to the completion of development at about 3 years. Thus, there is an assumption that insufficient intake of iron ions in the early development of primary teeth would lead to a higher rate of caries in primary teeth. Besides, inflammatory response that accompany ECC produce uncertain cell factors, which restrain the production of hemoglobin and further reduce the iron level [30].

In addition, a longer duration of breastfeeding was significantly associated with IDA and ECC. The content of iron in breast milk is insufficient for the infants’ growth and development [29]. Moreover, prolonged breastfeeding may interfere with saliva flow in the children and hinder their self-cleaning function, increasing the risk for IDA and ECC. The American Academy of Pediatrics suggests that exclusively breastfed infants should be supplemented with 1 mg/kg of iron daily from the fourth month until the time when the infant can consume sufficient iron-containing foods to reduce nutritional deficiencies [31]. However, most parents usually do not provide iron supplements, which might be related to the increased risk of cognitive development impairment [32].

There was wide public awareness of IDA, the most important type of anemia in children aged 2 years or under [33]. The age between 6–23 months is critical for children's growth and development. This is the period where iron reserves from a mother's pregnancy are completely consumed and the body's demand for iron is very high. Insufficient supply of nutrients in the body at this period leads to increased risk of IDA [34, 35]. Besides, unhealthy eating habits such as food avoidance and partial eating during this period would likely lead to future IDA, consistent with the findings in our questionnaire survey. Children aged 2 years or less with an IDA history were shown to be more likely to develop IDA at 4–6 years old, and the body is more prone to ECC in a state of nutritional deficiency.

Our study also showed that children’s intake of iron supplements reduces ECC and IDA, but it is not clear whether intake of iron supplements improves the nutritional status or increases the resistance to cariogenic bacteria [36, 37]. Besides, whether iron supplements can directly supplement iron and thus inhibit enamel demineralization is yet to be determined [28]. Excessive use of iron preparations in children increases the risk of development of tooth black stains and affects the appearance of teeth [38]. Therefore, children should take iron preparations in an appropriate amount.

On the other hand, data has shown that meat contains a large amount of heme iron and other forms of iron ions that can be well absorbed. Limited or lack of consumption of meat food increases the probability of IDA [39]. In contrast, our study showed that there was no significant association between the frequency of meat intake and IDA. Seafood in Qingdao is rich in iron, and the consumption of seafood can supplement iron in children [40].

IDA was shown to fuel ECC in severe cases of primary teeth caries in preschooler's in Qingdao. Factors that influence IDA, such as IDA history at 2 years old or less, mother's history of IDA during pregnancy, breastfeeding until 2 years old, and lower family income were shown to also cause ECC. Similarly, intake of iron preparations can inhibit the occurrence of ECC and IDA. Our study showed that the factors that influence IDA can lead to caries. However, there is need for in vivo and in vitro experiments to verify whether IDA has a significant effect on the development of children's primary teeth.

Our study was limited by use of limited items that were involved in the questionnaire to perform a comprehensive analysis between IDA-associated factors and ECC. The reliability of the questionnaire survey depended on the parent's memory and could trigger a recall or reaction bias. Although the sample size was sufficient for the survey, there are 10 districts and counties in Qingdao, thus the two districts that were randomly selected with the PPS method may not completely represent the whole city. Moreover, those children who did not cooperate with the doctors were excluded and could have influenced the outcome of this study. Finally, development of ECC or IDA takes a long time, which is influenced by many factors. Therefore, a longitudinal study will better illustrate the factors that mediate ECC and IDA.

## Conclusion

Taken together, our data demonstrated that factors such as low family income, IDA history in children 2 years old or younger, history of IDA during pregnancy, breastfeeding for 2 years and intake of iron supplements were significantly associated with ECC and IDA.

## Supplementary Information

**Additional file 1**. The questionnaire about Iron deficiency anemia (IDA) influencing factors in Qingdao, China (Version for Children’s Guardians).

## Data Availability

The datasets used and/or analyzed in this study are available from the corresponding author on reasonable request.

## Abbreviations

ECC: Early childhood caries
IDA: Iron deficiency anemia
Dmft: Decayed, missing and filled teeth in primary teeth
ID: Iron deficiency
PPS: Probabilistic proportional sampling
RMB: Renminbi
ICC: Intraclass Correlation Coefficient
