# Supplementary material for: Iron deficiency anemia associated factors and early childhood caries in Qingdao
Source: BMC Oral Health. 2022 Mar 31;22:104. doi: 10.1186/s12903-022-02127-z (PMC8973523; doi:10.1186/s12903-022-02127-z)
Supplement: Supplementary file 1 — Additional file 1. The questionnaire about Iron deficiency anemia (IDA) influencing factors in Qingdao, China (Version for Children’s Guardians). [file 12903_2022_2127_MOESM1_ESM.docx]

**The questionnaire about Iron deficiency anemia (IDA) influencing factors in Qingdao, China (Version for Children’s Guardians)**

Subject’s ID: Name of the subject:

Subject’s birthdate (mm/dd/19yy): Sex (F or M):

Date of survey: No. of the interviewer:

Please draw a “√” before the corresponding option, only one answer for all the questions.

1.Did the mother have an IDA history during pregnancy?

1) Yes 2) No

2. What is your annual family income（ten thousand RMB）?

1) <5 2) 5～10 3) 10-20 4) >20

3. How often does your child have meat below?

1)rarely or never 2)once to three times a week 3) once a day or more

4. Did the child have an IDA history at age 2 or below?

1) Yes 2) No

5. Did the child remain breastfeeding up to age 2?

1) Yes 2) No

6. Did your child ever consume iron supplements?

1) Yes 2) No
